# Correlation between vaginal microbiota and different progression stages of cervical cancer

**DOI:** 10.1590/1678-4685-GMB-2020-0450

**Published:** 2022-03-18

**Authors:** Bing Wei, Yi Chen, Tingyan Lu, Wenjiao Cao, Zhenhua Tang, Haiou Yang

**Affiliations:** 1 Shanghai Jiao Tong University, School of Medicine, International Peace Maternity and Child Health Hospital, Shanghai, China.; 2 Shanghai Key Laboratory of Embryo Original Diseases, Shanghai, China.

**Keywords:** HR-HPV, LSIL, HSIL, cervical cancer, vaginal microbiota

## Abstract

The process from high-risk human papillomavirus (HR-HPV) infection to cervical cancer is a continuous and long-term process, but the pathogenesis of the whole process is not completely clear. Here, 59 Chinese women were engaged in this study, and divided into five groups: normal healthy group, HR-HPV infections group, low-grade intraepithelial neoplasia (LSIL) group, high-SIL(HSIL) group, and cervical cancer group. With the occurrence of HR-HPV infection and the development of cervical lesions, the diversity of vaginal microbiota species was increased, and the relative abundance of *Lactobacillus (L.)*, the dominant bacteria in maintaining vaginal microecological balance, was decreased gradually. In contrast, the abundance of *Actinobacteria* in the four disease groups was significantly higher than that in normal group. Furthermore *L. iners* may be related to the serious progression of cervical cancer. After analyzing the whole process, we found that *Gardnerella*(*G.*), *Atopobium*(*A.*) and *Dialister(D.)* have important effects on both persistent HR-HPV infection and the pathogenesis of cervical cancer. In addition, PICRUSt2 and KEGG results showed that the KEGG pathways enriched by the predicted genes of vaginal microbiota in cancer group included metabolic diseases, endocrine system and immune systems when compared with that in normal group. These findings may provide insights into the pathogenesis of cervical cancer, and help to improve the early detection and prevention of cervical precancerous lesions.

## Introduction

In recent years, female vaginal microbiota has attracted more and more attention from medical researchers. Microbial communities living in the reproductive tract seem to be involved not only in maintaining female reproductive tract health, but also in reproductive tract diseases, reproductive failure and pregnancy complications (Al-Nasiry et al., 2020). Cervical cancer is one of the most common cancers in women worldwide (Ginsburg et al., 2017). Persistent HR-HPV infection has been recognized as closely associated with squamous intraepithelial lesions (SILs) and cervical cancer (Munoz et al., 2003). Factors that alter the vaginal microbiota, have been identified as cofactors in the persistence of HPV infection, such as sexual intercourse, vaginal irrigation, inflammation of the vagina, or sexually transmitted infections (Clarke et al., 2012; Vriend et al., 2015; Łaniewski et al., 2018). Most women can recover from HR-HPV infection without developing cervical cancer due to the elimination of HR-HPV by their immune system (Insinga et al., 2011; Greer et al., 2016). The vaginal microbiota in healthy women of reproductive age is dominated by *Lactobacillus (L.)*, which is known to produce lactic acid to maintain low vaginal pH (< 4.5), and this is the first line of defense against pathogenic agents (Boskey et al., 2001). Additionally, the vaginal microbiota is able to produce specific metabolites and bacteriocins which synergize or inhibit each other (Aroutcheva et al., 2001; McMillan et al., 2011). This suggests that vaginal microenvironment plays an important role in women’s reproductive health. 

Recently, next-generation sequencing of barcoded 16S rRNA has broadened our understanding of vaginal microbiota, and helps to better study the relationship between the composition of vaginal bacterial community and disease. Previous studies have proposed that abnormal vaginal microbiota are classified into five main communities dominated by *L. crispatus, L. iners, L. gasseri* and *L. jensenii*, while the other community is characterized by a decrease in *L.* spp. and an increase in bacterial diversity (Brotman et al., 2014). However, among them, the vaginal microbiota dominated by *L. iners* is more permissive (for example in receiving potential pathogens in the vaginal ecosystem) than others. And it is associated with microbiological disorders, and increases the risk of cervical and other female genital tract diseases (Mitra et al., 2015; Stokholm et al., 2018; McKinnon et al., 2019). In addition, several studies have shown that the microbiota can promote cellular metabolism and immune disorders, and play an important role in the development of cancer (Banerjee et al., 2018; Porter et al., 2018). Although these results do not explicitly state a causal relationship, they suggest a link between microbiota signatures and cancer in different organs. Recently, some studies have shown that vaginal microbiota are associated with HPV infection, as well as CIN and CC (Di Paola et al., 2017; Kyrgiou et al., 2017; McKee et al., 2019). But there are some differences in reported conclusions. Some epidemiological reports indicate that the vaginal microbiota of women vary by race and region (Łaniewski et al., 2018; Noyes et al., 2018). In this study, we explored the characteristics of vaginal micmbiome in normal, HR-HPV-positive, low-grade squamous intraepithelial lesion (LSIL), high-grade squamous intraepithelial lesion (HSIL) and cervical cancer women. And the correlation between vaginal microbiota and different progression stages of cervical cancer were further analyzed.

## Subjects and Methods

### Study design and sample collection

Premenopausal nonpregnant women from department of Cervical Disease of International Peace Maternity and Child Health Hospital, Shanghai Jiao Tong University School of Medicine were recruited. The exclusion criteria for the study are as follows: 1) Subjects having immunosuppressive diseases; 2) Subjects using antibiotic within the last 7 days; 3) Subjects currently having a sexually transmitted infection or vaginal infection; 4) Subjects using suppositories, vaginal applied medications and douching substances within 48 hours prior to the visit; 5) Subjects having had sexual intercourse less than 48 hours prior to the visit; 6) Subjects having a previous history of cervical treatment.

The total of 59 subjects were divided into five groups according to the HPV infection status and Histology of colposcopy-directed biopsy samples, including normal controls (normal), HR-HPV infection (HPV), low-grade squamous intraepithelial lesion (LSIL), high-grade squamous intraepithelial lesion (HSIL) and cervical cancer (cancer). HPV status was determined by the Linear Array HPV Genotyping Tests (Roche, Indianapolis, IN), and divided into two groups: HPV-positive (including HPV 16/18 genotypes) and HPV-negative controls (normal). 

This study was approved by the Ethics Committee of International Peace Maternity and Child Health Hospital (Approval number: GKLW2017-139). Vaginal swabs were collected by clinicians according to the clinical standards, and patient information and experiments were performed according to approval standards. All samples were sent to the laboratory immediately after collection and saved at −80 °C for subsequent DNA extraction.

### Bacterial DNA extraction

Total DNA of samples were extracted according to the Fast DNA SPIN extraction kits (MP Biomedicals, Santa Ana, CA) instruction. DNA concentration and purity were detected by NanoDrop2000, and DNA quality was tested using 1% agarose gel electrophoresis. DNA was stored at -20 °C before further analysis.

## DNA amplification and sequencing of barcoded 16S RNA gene fragments

PCR amplification of the V3-V4 regions of the bacterial 16S rRNA gene was performed using 338F (5’-ACTCCTACGGGAGGCAGCAG-3’) and 806R (5’-GGACTACHVGGGTWTCTAAT-3’) primers. The PCR reagent system contained 2 μL of 2.5 mM dNTPs, 4 μL of 5× Fast *Pfu* Buffer, 0.4 μL of Fast *Pfu* Polymerase, 0.8 μL of each primer (5 μM), and 10 ng of template DNA. PCR amplified procedure was as follows: 95 °C for 3 min, followed by 27 cycles at 95 °C for 30 s, 55 °C for 30 s, 72 °C for 30 s and a final extension at 72 °C for 10 min (PCR instrument: ABI GeneAmp9700).

PCR products were separated with 2% agarose gel, and purified using an AxyPrep DNA Gel Extraction Kit (Axygen Biosciences, Union City, CA, USA). Quanti Fluor - ST (Promega, USA) was used for quantitative detection. PE 2^*^300 library was constructed from purified amplified fragments according to Illumina MiSeq platform (Illumina, San Diego, USA) standard operating procedures. Steps for library construction: (1) Connecting “Y” connector; (2) Using magnetic beads to remove self-connecting segments; (3) Enriching library templates by PCR amplification; (4) Producing single stranded DNA fragments by sodium hydroxide denatures. And the Illumina MiSeq PE300 platform (Illumina, San Diego, USA) was used for sequencing.

### HPV genotyping

HPV 16/18 genotyping was performed using the Roche Linear Array HPV Genotyping Test (Roche, Basel, Switzerland) according to manufacturer’s guidelines. A clinically validated *in vitro* polymerase chain reaction (PCR) assay was used to identify HPV-16, -18 and 12 other HR HPV subtypes (31, 33, 35, 39, 45, 51, 52, 56, 58, 59, 66, 68). 

### Sequence analysis

The original sequencing was controlled by Trimmomatic software (Bolger et al., 2014) and spliced with FLASH software (Quan et al., 2019). Sequences with a length of <150 bp and an average Phred score of <20 were filtered out. Sequences that had ambiguous bases were removed, and sequences that had mononucleotide repeats shall be more than 10. Sequences that could be spliced were removed using UPARSE software (version 7.1), according to the similarity of 97% to OTU sequence clustering. Chimeras were removed using UCHIME software. For each sequence, the RDP classifier was used to annotate the species classification, and the comparison was made to the Silva database (SSU123), with a threshold value of 70%.

### Statistical analysis

Quantitative insights into microbial ecology (QIIME) software and R packages were used to analyze the sequence information. Bacterial diversity was assessed using the alpha diversity: Shannon and Simpson diversity index. The difference of alpha diversity index between different groups was analyzed using Student’s *t*-test (p < 0.05) (Huang et al., 2018). The R language vegan package was adopted to perform species segmentation and aggregation, which was represented on the community heat map. In addition, we used unweighted principal component analysis to carry out UniFrac distance measurement by PCA (principal component analysis), and we observed the interaction between bacterial communities in different groups by R packages. Partial Least Squares Discriminant Analysis (PLS-DA) was used to analyze environmental factors affecting microecology. LEfSe (http://huttenhower.sph.harvard.edu/galaxy/) was employed to detect differentially abundant genera between groups for biomarker discovery (Segata et al., 2011). By calculating the Pearson correlation coefficient, the cooccurrence patterns of the 30 most abundant taxa in different groups are discussed. Using data from the Kyoto Encyclopedia of Genes (KEGG) dataset, PICRUSt made predictions about the functional composition of the bacterial community (Langille et al., 2013). 

## Results

### Subjects information

In the current study, 59 eligible women were recruited and classified into five groups: normal controls (normal; n = 10), HR-HPV infection (HPV; n = 13), low-grade squamous intraepithelial lesion (LSIL; n = 15), high-grade squamous intraepithelial lesion (HSIL; n = 10) and cervical cancer (cancer; n = 11) (Table 1 ). In total, 2,866,307 reads were acquired from 59 samples, with an average number of 47,771±12,155 reads per sample. Following removal of rare OTUs and single tons, in this study, a total of 333 taxa were observed in the vaginal microbiota.

**Table 1 -:** Characteristics of study participants and hrHPV status, P values were calculated using ANOVA for continuous variables.

	n	normal (n = 10)	HPV+ ( n = 13)	LSIL ( n = 15)	HSIL ( n = 10)	Cancer ( n = 11)	P value
age(mean(SD))	59	38.50(5.68)	37.08(7.05)	38.33(7.35)	40.50(5.95)	43.45（5.50）	0.19
HPV status							
HPV 16 positive		7（53.85）	5（33.33）	6（60.00）	4（36.36）	0.65	
HPV 18 positive		7（53.85）	2（13.33）	1（10.00）	2（18.18）	0.28	
other high-risk		3（23.08）	8（53.33）	6（60.00）	6（54.55）	0.47	
Single high-risk		9（69.23）	9（60.00）	7（70.00）	4（36.36）	0.79	
Multiple high-risk		4（30.77）	2（13.33）	3（30.00）	2（18.18）	0.79	

### Bacterial community characterization

**Figure 1-:**
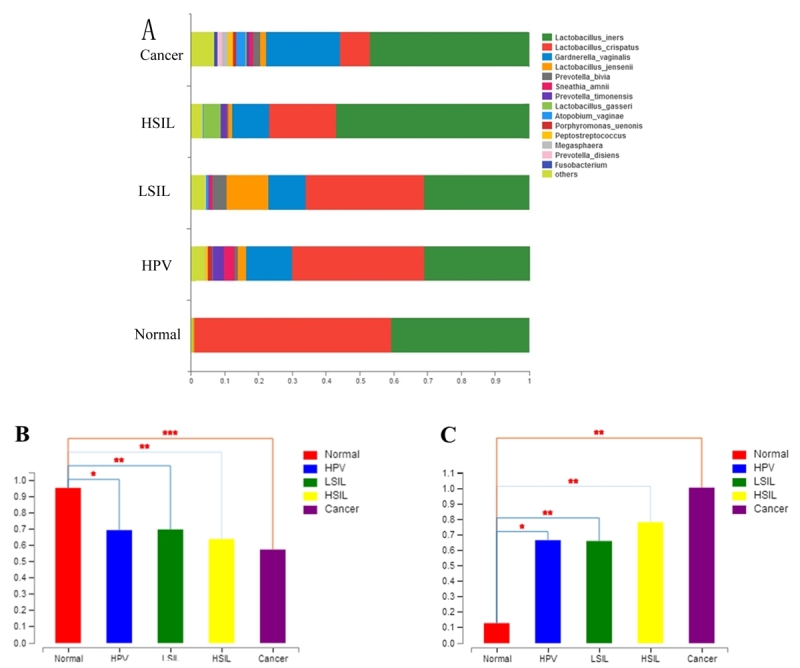
The comparisons of the five groups in vaginal microbiota. (A) Bar chart describing the difference in bacterial diversity between the normal, HPV, LSIL, HSIL and cancer groups. (B) Simpson index of OTU level and P values calculated using Student`s *t*-test (P ≤ 0.05 was marked as ^*^; 0.001 < P ≤ 0.01 was marked as ^**^; P ≤ 0.001 was marked as ^***^). (C) Shannon index of OTU level and P values calculated using Student`s *t*-test (P ≤ 0.05 was marked as ^*^; 0.001 < P ≤ 0.01 was marked as ^**^; P ≤ 0.001 was marked as ^***^).

In this study, the compositions of vaginal microbiota in the five groups were investigated (Figure 1A and Table S1). The majority of vaginal bacteria belonged to six major phyla: *Firmicutes*, *Bacteroidetes*, *Fusobacteria*, *Actinobacteria*, *Proteobacteria* and *Tenericutes*. Of these, the number of *L. spp.* belonging to *Firmicutes* was the most abundant (78.04%) in all five groups. In addition, the species diversity of vaginal microbiota in the four disease groups (HR-HPV, LISL, HISL and cancer groups) was increased compared with that in normal group. Among them, the species diversity of vaginal microbiota in the cancer group was further increased, and the most significant change at the genus level was the large increase in the relative abundance of low-abundance bacteria or even unknown bacteria, indicating that the community structure of vaginal microbiota was disorganized.

The *L. spp.* abundance in the HR-HPV infection, LSIL, HSIL and cervical cancer group was significantly decreased compared with the normal group (Figure 1B). In contrast, the abundance of *Actinobacteria* (mainly *G.* and *A*.) in the four disease groups was higher than that in the normal group, and cancer group had the highest abundance (Figure 1C). The increased abundance of *Bacteroidetes* (mainly *Prevotella*) and *Fusobacteria* (mainly *Sneathia*) was also positively correlated with disease progression, but the increase was not as significant as that of *Actinobacteria*.

The Simpson diversity index and Shannon diversity index were used to evaluate the α-diversity of the bacterial community. The diversity index calculated by *t*-test indicated significant differences between the normal control group and disease group; the diversity became more significant with the severity of the disease (HPV-normal p=0.01851; LSIL-normal p=0.003303; HSIL-normal p=0.007463; cancer-normal p= 0.0008941), but there were no statistically significant differences between the disease groups (Figure 1B and Table S2). 

**Figure 2 -:**
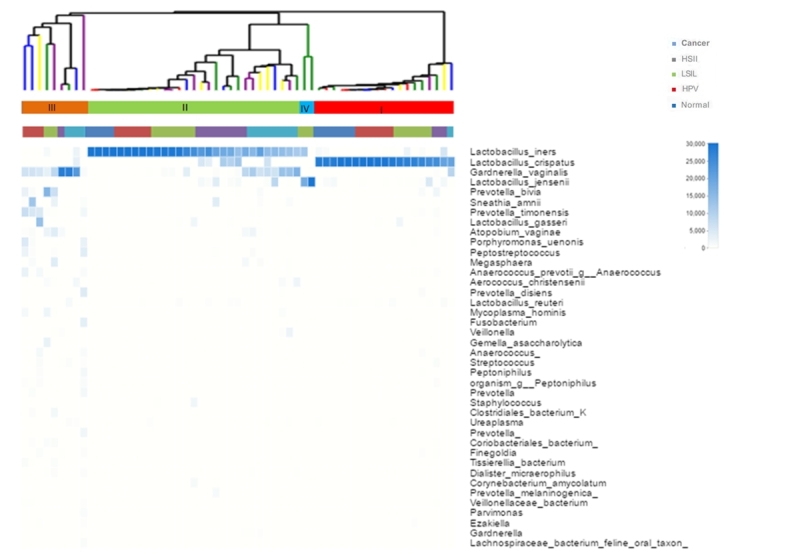
Heat map analysis, each vertical line represents a sample. Relative abundance is shown in blue and white: blue indicates a high proportion, and white indicates a low proportion. Five groups (normal, HPV, LSIL, HSIL, cancer) were distributed in different CSTs with different colors.

Heat map analysis of the sequence data using nearest neighbor linkage at the species level identified four major vaginal microbiota community state types (CSTs) (Figure 2 and Table S3). Among all detected strains, *L*. *spp.* was the most abundant microorganism. In the identified *L*. species, *L. iners* and *L. crispatus* were the most abundant (39.7% for *L. iners* and 32.3% for *L. crispatus*) in this study. In addition, *L. jensenii* were identified in 4.3% of subjects, and *L. gasseri* is was even rarer, with only 0.9%. 

The frequency of CST I (*L. crispatus*-dominant) decreased with increasing disease severity (normal = 6/10, 60%; HPV =5/13, 38%; LSIL = 4/15, 27%; HSIL = 2/10, 20%; cancer = 1/11, 9%). Conversely, the rate of CST II (*L. iners*-dominated) was associated with an increase in HSIL and cancer group compared with the normal group (normal = 4/10, 40%; HPV =5/13, 38%; LSIL = 6/15, 40%; HSIL = 7/10, 70%; cancer = 7/11, 64%). None of normal group samples were found in CST III (*L*.-depleted), and the cancer group accounted for the largest proportion (HPV =3/13, 23%; LSIL = 2/15, 13%; HSIL = 1/10, 10%; cancer = 3/11, 27%). Due to the small sample size, two of the CST IV samples were LSIL, which could not explain the relationship between CST IV and disease. 

**Figure 3-:**
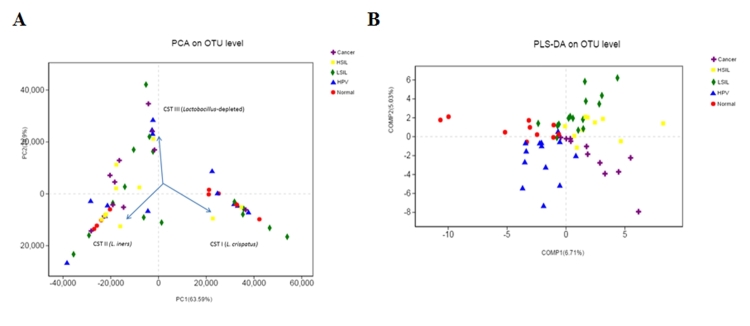
Principal component analysis (PCA) of species sequence data and partial Least Squares Discriminant Analysis (PLS-DA) analysis of the high-dimensional data. (A) Principal component analysis (PCA) showed that the vaginal microbiota identified three major clusters *L. crispatus* (CST I), L. iners (CST II), and depleted of L. spp. with high diversity (CST III). (B) Partial least square discriminant score plot of vaginal microbiota between the normal, HPV, LSIL, HSIL and cancer groups.

Principal component analysis (PCA) of species sequence data was used to evaluate the vaginal community structure in the context of disease grade (normal, HPV, LSIL, HSIL and cancer) (Figure 3A). Compared with the results of the heat map analysis, the conclusion was roughly the same; however, due to the low abundance of *L. jensenii*, the three main clusters were identified, which represented samples dominated by species with higher diversity, and the other two were *L. crispatus* and *L. iners*.

Then, we used Partial Least Squares Discriminant Analysis (PLS-DA) to analysis the high-dimensional data (Figure 3B). The bacterial communities in the normal, HPV, LSIL, HSIL and cancer groups clustered separately, which indicates that the vaginal microbiota composition of the serious progression of cervical cancer and healthy people is significantly different. The normal group and HPV infection group were in the second and third quadrants, respectively. The LSIL and HSIL groups were close to each other, both of which were in the first quadrant and partly intersect, indicating a high correlation between the vaginal microbiota of the two disease stages. The cancer group was located in the fourth quadrant, and it showed the maximum distance from the normal group, indicating a more significant difference between the two groups. In addition, it can be seen from the discrete distribution of sample points in the PLS-DA analysis that the vaginal microbiota composition of different patients varies greatly.

### Identification of vaginal microbiota composition markers

**Figure 4 -:**
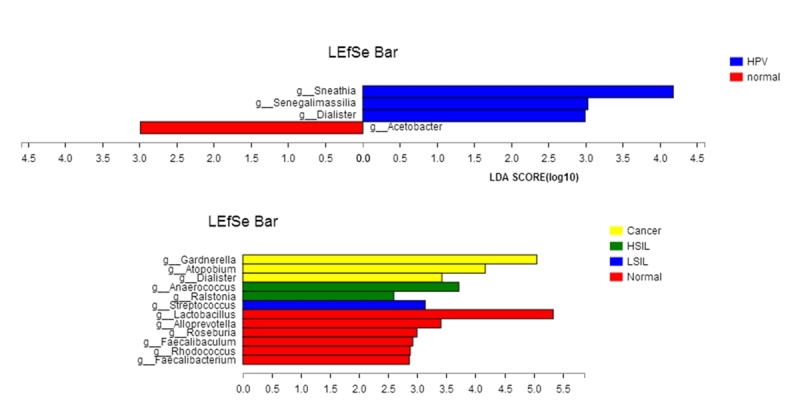
Linear discriminate analysis effect size (LEfSe) was performed between different disease stages. (A) According to HPV infection, LEfSe was used to detect the difference in relative abundance of microbiota. (B) According to cervical intraepithelial neoplasia status, LEfSe was used to detect difference in microbiota relative abundance. Only taxa with LDA scores of >2 are presented.

The linear discriminant analysis (LDA) effect size (LEfSe) model was used to identify the differences in microbiota composition, which may be associated with increased disease severity. At the genus level, three taxa exhibited significantly higher abundances in the HPV group than in the normal group, including *D.*, *Sneathia*, and *Senegalimassilia*. The *Acetobacter* taxa were remarkably prevalent in the controls (Figure 4A).

There were significant differences in the community compositions between LSIL, HSIL, and cervical cancer groups at the genus level. There were six significant genera in the normal group: *Faecalibaculum*, *Rhodococcus*, *Roseburia*, *L.*, *Faecalibacterium* (p<0.01) and *Alloprevotella* (p<0.04). In the LSIL group, *Streptococcus* was significantly overrepresented, and in the HSIL group, the highest abundance was *Ralstonia* (p<0.01) and *Anaerococcus* (p<0.03). *G.* (p<0.01), A. and D. (p<0.03) were the predominant genera in the cancer groups. These bacteria with significant differences between groups can be suggested as potential biomarkers for disease diagnosis (LDA> 2, p<0.05) (Figure 4B).

### Microbiota functional predictions in cancer and normal groups

**Figure 5-:**
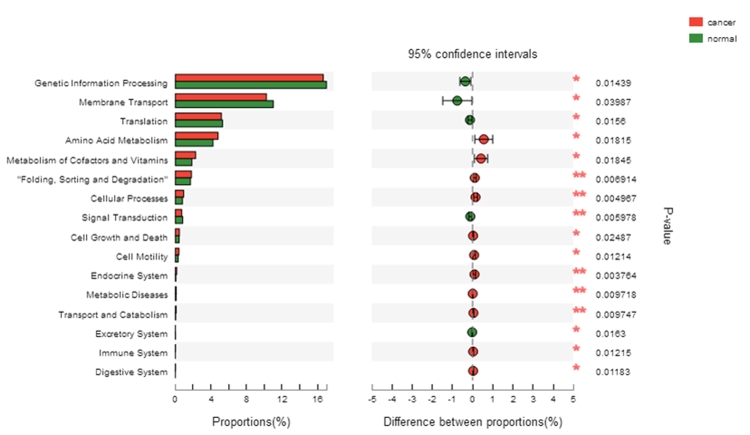
PICRUSt infers the cellular functions of bacterial communities in cervical cancer.

PICRUSt was used to infer the bacterial functional metagenomic content of each sample and to classify the inferred genes into the level 2 KEGG pathways. The vaginal microbiota of the normal group was characterized by signal transduction, over representation of membrane transport, excretory system, genetic information translation and processing, all of which may be related to the normal physiological metabolism of bacteria and cells. In contrast, compared with the normal group, the cancer group showed highlighted metabolic diseases, endocrine system, immune system, cellular processes, cell growth and death, cell motility, transport and catabolism as well as “folding, sorting and degradation” (Figure 5).

## Discussion

HR-HPV infection is a recognized risk factor for squamous intraepithelial lesions (SILs) and cervical cancer occurrence. In addition, there are many other factors contributing to cervical cancer development. In this study, we compared the structure and diversity of vaginal microbiota in normal, HR-HPV infection, LSIL, HSIL and cervical cancer groups, and analyzed the differences in bacterial composition and gene function during the development of cervical cancer. 

We found that the species diversity and community structure of vaginal microbiota were different among normal, HR-HPV groups, and groups with different grades of cervical lesions. With the occurrence of HR-HPV infection and the development of cervical lesions, the diversity of vaginal microbiota species was increased, and the community structure became more complex and disordered. In addition, the relative abundance of *L. spp.*, the dominant bacteria in maintaining vaginal microecological balance, was decreased gradually with the occurrence of HR-HPV infection and the development of cervical lesions. Among them, the change was most pronounced in the cancer group. In contrast, the abundance of *Actinobacteria* (mainly *G.* and *A*.) in the four disease groups was significantly higher than that in normal group. Among them, the change was also most pronounced in the cancer group. With the development of cervical lesions, the relative abundance of low-abundance bacteria and even unknown bacteria in vaginal microbiota was increased, indicating that the imbalance of vaginal microflora became more and more serious.

The vaginal microbiota in healthy women of reproductive age is dominated by *L*., which is known to produce lactic acid to maintain low vaginal pH (< 4.5), and this is the first line of defense against pathogenic agents (Boskey et al., 2001). In this study, *L. iners* and *L. crispatus* were the most abundant vaginal microbial communities. *L. jensenii* was the third abundant communitiy in *L*. *spp*. While *L. gasseri* content was only 0.9%. This phenomenon of only a small amount of *L. jensenii* and even less or no *L. gasseri* has also been found in Asian women in other studies (Lee et al., 2013; Huang et al., 2018). The characteristic seems to be unique to Asian women, as it differs from the vaginal microbial communities in European and American women that the content of *L. gasseri* is higher than that of *L. jensenii* (Brotman et al., 2014). These differences may be related to racial and regional differences, such as genetic and living conditions, innate and adaptive immune systems, ligands on epithelial cell surfaces, as well as the composition and quantity of vaginal secretions (Ravel et al., 2011; Anahtar et al., 2015; Dareng et al., 2016).

This study further found that the distribution of CSTs in normal group was different from the other disease groups. The abundance of *L. crispatus* was decreased in disease groups compared with normal group. While the frequency of CST III (*L*.-deficient) was increased during the disease progression. In this study, *L. spp.* in the HSIL group was slightly higher than that in the LSIL group, but the proportion of *L. iners* in *L. spp.* in the HSIL group was significantly higher than that in the LSIL group. The *L. spp.* content in the cancer group was reduced, while the proportion of *L. iners* continued to be increased, accounting for 80.44% of the *L. spp*.. The role of *L. iners* in the healthy cervix is unclear, since it was detected both in normal and disease groups (Petrova et al., 2017). *L. iners* is more common than *L. crispatus* in patients with vaginal dysbiosis caused by HSV-2 and HIV infection (Borgdorff et al., 2014). Some studies have shown that there are two isoforms of lactic acid, D-lactic acid and L-lactic, but they act differently in microbial. D-lactic acid level has been inversely associated with the ability of HIV to transverse cervicovaginal mucus. *L. crispatus* produces both the two isoforms, whereas L. iners produces only L-lactic acid (Nunn et al., 2015; Norenhag et al., 2020). Most *L*. *spp.* can produce H2O2 with antibacterial properties, except *L. iners* (Aroutcheva et al., 2001; Reimers et al., 2016), indicating that *L. iners* may be related with disease. Our results are consistent with these findings. The rate of CST II (*L. iners*-dominated) was increased in HSIL and cancer group compared with that in normal group. *L. iners* may also related to the serious progression of cervical cancer. In addition, the analysis of PCA and PLS-DA further confirmed the significant difference between the vaginal microbiota in disease group and the normal group. As a result, vaginal bacterial dysbiosis may have existed in the development of cervical cancer. 

Based on LEfSe analysis, some potential biomarkers related to different disease stages were found. *D.*, *Sneathia* and *Senegalimassilia* were significantly overrepresented in HPV group. *Streptococcus* were the most representative in LSIL. Whereas *Anaerococcus* and *Ralstonia* were significantly enriched in HSIL samples. Three other BV-associated bacteria, *G.*, *D*. and *A*., were also observed to be markers of cancer. Previous studies have identified *Sneathia spp.* as a microbiological marker for HPV infection, cervical neoplasm and increased HIV acquisition (Di Paola et al., 2017; Łaniewski et al., 2018; McClelland et al., 2018). In our study, we further analyzed the vaginal microbiota of HR-HPV infection, SIL and cervical cancer, and found that *Sneathia spp.* was more abundant in HR-HPV infection women than in patients with cervical cancer. To this extent, we confirmed that cervical disease was correlated with distinct bacterial combinations, which also changed with disease status. In addition, these specific bacteria were present in HR-HPV infection and CIN severity. We can assume that they contribute an important coordinating role in clearing HR-HPV infection or facilitating the transition from normal cytology to cervical squamous intraepithelial lesion status. 

Based on the 16S rRNA sequence information, PICRUSt was used to infer the function of the bacterial community, and further analyzed the differences between normal control and cervical cancer groups. The results showed that changes in bacterial abundance and composition could lead to significant changes in gene functional expression, especially in metabolic diseases, endocrine system, and immune system, which might be the factors of the development of cervical lesions.

A major strength of our study is that we have analyzed the vaginal microbiota of Chinese women from HR-HPV infection to precancerous lesions and further to cervical cancer. There are still some limitations, including a relatively modest sample size and uncollected pH data. In summary, our work provides evidences for elucidating the complex relationship between vaginal microbiota composition, HR-HPV infection and cervical intraepithelial neoplasia. We also identified bacterial biomarkers to distinguish HR-HPV infection, LSIL, HSIL and cervical cancer. This may improve our understanding of the role of the bacterial microenvironment in HPV persistence, CIN development and cancer progression. The relationship between vaginal microbiota and functional prediction may provide insights into the pathogenesis of cervical cancer. These specific bacteria will improve women’s health status while helping to improve the early detection and prevention of cervical precancerous lesions. In future studies, we will further study the relationship between vaginal bacteria and the tumor microenvironment.

## Supplementary material

The following online material is available for this article:

Table S1 - Bar chart describing the difference in bacterial diversity between the normal, HPV, LSIL, HSIL and cancer groups.

Table S2 - Statistical analysis of the differences between groups.

Table S3 - Clusters distribution of all the samples using heat-map analysis at the species level.
