# Supplementary material for: Correlation between vaginal microbiota and different progression stages of cervical cancer
Source: Genet Mol Biol. 2022 Mar 18;45(2):e20200450. doi: 10.1590/1678-4685-GMB-2020-0450 (PMC8967114; doi:10.1590/1678-4685-GMB-2020-0450)
Supplement: Table S1 - [file 1415-4757-GMB-45-2-e20200450-s1.pdf]

Genetics and Molecular Biology
https://doi.org/10.1590/1678-4685-GMB-2020-0450

Supplementary Material to "Correlation between vaginal microbiota and different progression stages of cervical cancer"

Table S1 - Bar chart describing the difference in bacterial diversity between the normal, HPV-pos, LISL, HSIL and cancer groups.

ID	Lactobacillus_iners	Lactobacillus_crispatus	Gardnerella_vaginalis	Lactobacillus_jensenii	Prevotella_bivia	Sneathia_amnii	Prevotella_timonensis	Lactobacillus_gasseri	Atopobium_vaginae	Porphyromonas_uenonis	Peptostreptococcus	Megasphaera	Prevotella_disiens	Fusobacterium	others
Cancer	14125.0909	2623.2727	6517.6364	525.1818	626	361.2727	189.5455	166.8182	793.4545	269.6364	453.4545	494.1818	402.2727	326.3636	2280.817
HSIL	16764.8	5992.9	3256.9	368.9	98.9	45.5	509.7	1552	11.1	3	5.5	56.5	10.5	0.5	1139.6
LSIL	9290.1333	10537.2667	3301.2	3740.0667	1258.2	271.9333	76.0667	14	182.7333	4.6667	5.8667	0	43.4667	1.9333	1333.1343
HPV	9477.6923	11866.9231	4141.2308	723.1538	263.1538	993.5385	956.8462	0.2308	68.2308	409.3846	217	67.2308	0.1538	0.2308	982.7678
Normal	12302.3	17579.4	3.9	113.9	1.3	0	5.4	19.8	0.1	0	0.1	0	0.1	0	197
