# Supplementary material for: Correlation between vaginal microbiota and different progression stages of cervical cancer
Source: Genet Mol Biol. 2022 Mar 18;45(2):e20200450. doi: 10.1590/1678-4685-GMB-2020-0450 (PMC8967114; doi:10.1590/1678-4685-GMB-2020-0450)
Supplement: Table S2 - [file 1415-4757-GMB-45-2-e20200450-s2.pdf]

Genetics and Molecular Biology
https://doi.org/10.1590/1678-4685-GMB-2020-0450

Supplementary Material to “Correlation between vaginal microbiota and different progression stages of cervical cancer”

Table S2 - Statistical analysis of the differences between groups.

Estimators	Pvalue(HSIL-LSIL)	Pvalue(HPV-HSIL)	Pvalue(Cancer-HSIL)	Pvalue(HPV-LSIL)	Pvalue(Cancer-HPV)	Pvalue(Cancer-LSIL)	Pvalue(LSIL-Normal)	Pvalue(HSIL-Normal)	Pvalue(HPV-Normal)	Pvalue(Cancer-Normal)
shannon	0.5854	0.6976	0.4987	0.983	0.3114	0.1992	0.002566	0.003731	0.03971	0.004151
simpson	0.5778	0.6698	0.6177	0.967	0.3554	0.2537	0.003303	0.002724	0.01851	0.000894
