# Supplementary material for: Correlation between vaginal microbiota and different progression stages of cervical cancer
Source: Genet Mol Biol. 2022 Mar 18;45(2):e20200450. doi: 10.1590/1678-4685-GMB-2020-0450 (PMC8967114; doi:10.1590/1678-4685-GMB-2020-0450)
Supplement: Table S3 - [file 1415-4757-GMB-45-2-e20200450-s3.pdf]

Genetics and Molecular Biology
https://doi.org/10.1590/1678-4685-GMB-2020-0450

Supplementary Material to “Correlation between vaginal microbiota and different progression stages of cervical cancer”

Table S3 - Clusters distribution of all the samples using heat-map analysis at the species level.

Species	N78	N273	N278	N206	N3	N21	N20	N5	N14	N252	N277	N30	N196	N285	N279	N270	N1	N13	N283	N113	N274	N268	N9	N227	N2	N38	N22	N144	N7	N16	N8	N236	N11	N19	N126	N10	N230	N18	N115	N275
Lactobacillus_iners	1144	0	42	2	2488	30032	30046	30222	30221	30199	30166	30125	30035	29791	29380	29516	26383	18783	20997	14959	14448	13172	11387	12050	90	1308	850	0	6	1	10	1	1	5	126	165	0	2418	592	9
Lactobacillus_crispatus	1	0	7	1	3374	0	0	2	0	0	2	0	0	0	0	1	1	11127	0	3	0	0	0	5	10	28487	28370	28574	30044	30064	29974	30104	30338	29891	29811	29211	28143	27610	26526	25121
Gardnerella_vaginalis	33	85	282	281	222	0	0	1	0	12	0	27	2	108	696	685	36	0	63	62	133	130	165	29	72	0	0	0	0	0	0	4	0	2	0	10	2	0	0	24

Species	N78	N2773	N2788	N2206	N3	N21	N20	N5	N14	N2252	N2277	N30	N1196	N2285	N2279	N2270	N1	N13	N2283	N1113
	07	22	20	73	78												31		04	79
Lactobacillus_jensenii	62	0	1	2	59	0	0	0	0	0	0	0	0	0	0	0	2	93	0	8813
Prevotella_bivia	16104	6798	0	0	0	0	1	3	0	7	2	0	0	3	4	0	0	5	959	0
Sneathia_amnii	0	2693	0	0	0	0	0	0	0	0	0	0	0	0	0	0	0	0	452	0
Prevotella_timonensis	288	145	58	0	0	0	4	0	0	4	0	0	0	0	4	0	0	0	105	1
Lactobacillus_gasseri	1	4	0	0	0	1	2	0	1	0	0	0	0	0	0	1	0	0	0	1

Species	N2274	N2268	N9	N2227	N2	N38	N22	N1144	N7	N16	N8	N2236	N11	N19	N1126	N10	N2230	N18	N1115	N2275
	45	51	23																	
Lactobacillus_jensenii	0	197	0	18124	29999	167	870	1417	36	25	36	90	55	40	69	58	156	99	211	4918
Prevotella_bivia	7	36	0	0	0	1	0	13	0	0	0	0	0	0	8	0	10	0	822	0
Sneathia_amnii	3	767	0	0	0	0	0	1	0	0	0	0	0	0	0	0	0	0	0	0
Prevotella_timonensis	32	26	0	0	2	0	0	2	3	0	0	2	0	0	46	0	1	0	206	0
Lactobacillus_gasseri	4	3	0	0	0	0	0	0	0	0	1	0	0	91	16	0	18	0	1	1

Species	N78	N273	N278	N206	N3	N21	N20	N5	N14	N252	N277	N30	N196	N285	N279	N270	N1	N13	N283	N113
Atopobium_vaginae	2410	4042	1073	0	0	0	0	1	0	0	0	0	0	0	0	11	0	0	85	0
Porphyromonas_uenonis	0	0	1	0	0	0	0	0	0	0	0	0	0	0	0	0	0	0	29	0
Peptostreptococcus	6	293	0	0	0	0	1	0	0	0	0	0	0	0	7	0	0	0	35	0
Megasphaera	0	4051	0	0	0	0	0	0	0	0	0	0	0	0	0	28	0	0	565	0
Anaerococcus_prevotii_g__Anaerococcus	106	1	15	0	0	0	0	0	0	2	1	0	0	0	6	0	0	0	9	0
Aerococcus_christensenii	769	181	0	0	15	0	0	1	0	0	0	0	0	0	0	7	37	0	27	0

Species	N274	N268	N9	N227	N2	N38	N22	N144	N7	N16	N8	N236	N11	N19	N126	N10	N230	N18	N115	N275
Atopobium_vaginae	25	78	0	0	0	0	0	0	0	0	0	0	0	0	0	0	461	0	0	0
Porphyromonas_uenonis	1	0	0	0	0	0	0	0	0	0	0	1	0	0	0	0	0	0	0	0
Peptostreptococcus	19	20	0	0	0	0	0	0	0	0	0	0	0	0	0	0	0	0	0	0
Megasphaera	0	116	0	0	0	0	0	0	0	0	0	0	0	0	0	0	0	0	0	0
Anaerococcus_prevotii_g__Anaerococcus	57	70	0	1	0	1	0	0	0	0	0	0	0	0	2	0	0	0	130	0
Aerococcus_christensenii	941	66	20	0	0	0	0	0	0	0	0	0	0	0	0	6	0	0	0	0

Species	N78	N273	N278	N206	N3	N21	N20	N5	N14	N252	N277	N30	N196	N285	N279	N270	N1	N13	N283	N113
Prevotella_disiens	3	0	0	0	460	0	0	0	0	2	0	0	0	0	11	0	0	0	9	0
Lactobacillus_reuteri	1	0	2	1088	1	0	0	0	0	2	0	0	141	0	0	0	1	1	0	1
Mycoplasma_hominis	2057	98	0	0	0	0	0	0	0	0	0	0	0	0	0	0	0	0	0	0
Fusobacterium	1	0	0	0	0	0	0	0	0	3	0	0	0	0	0	0	0	0	0	0
Veillonella	0	75	294	0	0	0	0	0	0	0	0	0	0	0	0	0	5	0	0	0
Gemella_asaccharolytica	28	0	0	0	0	0	0	0	0	0	0	0	0	0	0	0	0	0	1	0

Species	N274	N268	N9	N227	N2	N38	N22	N144	N7	N16	N8	N236	N11	N19	N126	N10	N230	N18	N115	N275
Prevotella_disiens	93	15	490	0	0	0	0	0	1	0	0	0	0	0	0	0	3	0	584	0
Lactobacillus_reuteri	0	0	63	1	0	15	51	60	99	1099	89	1	0	145	8	543	1	0	6	129
Mycoplasma_hominis	516	0	0	0	0	0	0	1	0	0	0	0	0	0	0	0	4	0	0	0
Fusobacterium	0	3	0	0	1	1	0	0	0	0	0	0	0	0	0	0	0	0	0	0
Veillonella	296	2202	0	0	0	0	0	0	0	0	0	0	0	0	0	0	0	0	0	0
Gemella_asaccharolytica	20	2	0	0	0	0	0	0	0	0	0	0	0	0	0	0	0	0	0	0

Species	N7879	N273	N278	N206	N3	N21	N20	N5	N14	N252	N277	N30	N196	N285	N279	N270	N1	N13	N283	N113	N274	N268	N9	N227	N2	N38	N22	N144	N7	N16	N8	N236	N11	N19	N126	N10	N230	N18	N115	N275
Anaerococcus_	0	13	11	0	0	2	1	0	0	1	3	0	0	0	10	0	0	2	1	0	9	6	0	0	0	0	0	1	0	0	0	0	0	0	4	0	0	0	394	0
Streptococcus	3	0	183	511	0	0	0	0	0	1	0	0	0	3	2	0	11	1	0	0	24	28	0	0	4	0	0	0	0	0	0	0	0	0	2	1	2	0	26	0
Peptoniphilus	0	0	6	0	0	0	1	0	0	0	0	0	0	0	2	0	0	0	1	0	11	3	0	0	0	0	0	0	0	0	0	0	0	0	1	0	0	0	98	0
organism_g__Peptoniphilus	0	1	34	0	0	0	4	1	0	1	1	0	0	0	3	0	0	0	1	0	22	32	1	0	1	0	0	0	0	0	0	0	0	0	9	0	0	0	39	0
Prevotella	0	0	3	0	0	0	1	0	0	0	0	0	0	0	1	0	0	0	17	0	1	3	0	0	0	0	0	0	0	0	0	0	0	0	5	0	0	0	0	0
Staphylococcus	0	1	1	0	1	0	3	0	0	0	1	0	0	0	2	0	1	2	0	0	2	10	1	0	0	0	2	0	0	1	0	0	0	0	2	0	1	0	0	0
Clostridiales_bacterium_K	0	1630	1	0	0	0	0	0	0	0	0	0	0	0	0	2	0	0	1	0	3	0	0	0	0	0	0	0	0	0	0	0	0	0	0	0	0	0	0	0

Species	N78	N273	N278	N206	N3	N21	N20	N5	N14	N252	N277	N30	N196	N285	N279	N270	N1	N13	N283	N113	N274	N268	N9	N227	N2	N38	N22	N144	N7	N16	N8	N236	N11	N19	N126	N10	N230	N18	N115	N275
Ureaplasma	21	10	0	456	300	4	0	20	0	4	0	32	17	313	42	5	12	0	0	25	11	66	1	0	0	94	2	3	4	0	0	4	0	2	21	155	0	0	21	2
Prevotella_	0	0	0	0	0	0	2	0	0	0	0	0	0	0	0	0	0	0	0	0	1	0	0	0	0	0	0	0	0	0	0	0	0	0	0	0	0	0	0	0
Coriobacteriales_bacterium_	753	617	0	0	6	0	0	0	0	0	0	0	0	0	0	0	0	0	17	0	26	0	0	0	0	0	0	0	0	0	0	0	0	0	0	0	0	0	0	0
Finegoldia	1	0	88	0	0	0	1	2	0	5	7	0	0	2	20	0	0	4	0	0	20	47	0	0	3	4	0	2	2	0	0	2	1	0	15	1	3	0	253	0
Tissierellia_bacterium	0	505	0	0	0	0	0	0	0	0	0	0	0	0	0	0	0	0	23	0	2	0	0	0	0	0	0	0	0	0	0	0	0	0	0	0	0	0	0	0
Dialister_micraerophilus	96	414	0	0	1	0	1	0	0	0	0	0	0	1	0	0	0	0	12	0	61	18	84	0	0	0	0	0	1	0	0	0	0	0	2	0	0	0	9	0
Corynebacterium_amycolatum	0	0	7	0	0	0	0	0	0	0	0	0	0	0	0	0	0	0	0	0	0	45	0	0	0	3	0	1	0	0	0	0	0	0	0	0	0	0	0	0
Prevotella_melaninogenica_	0	0	0	0	0	0	0	0	0	0	0	0	0	0	0	0	0	0	0	0	0	0	0	0	0	0	0	0	0	0	0	0	0	0	0	0	0	0	0	0

Species	N78	N273	N278	N206	N3	N21	N20	N5	N14	N252	N277	N30	N196	N285	N279	N270	N1	N13	N283	N113
Veillonellaceae_bacterium	16	44	1	0	0	0	0	0	0	0	0	0	0	0	1	0	0	0	4	0
Parvimonas	0	0	0	0	0	0	0	0	0	0	0	0	0	0	0	0	0	0	0	0
Ezakiella	0	0	4	0	0	0	0	0	0	0	0	0	0	0	1	0	0	0	0	0
Gardnerella	9	0	0	0	5	0	0	0	0	0	0	1	1	4	27	1	6	0	259	55
Lachnospiraceae_bacterium_feline_oral_taxon_	0	0	0	0	0	0	0	0	0	0	0	0	0	0	0	0	0	0	0	0

Species	N274	N268	N9	N227	N2	N38	N22	N144	N7	N16	N8	N236	N11	N19	N126	N10	N230	N18	N115	N275
Veillonellaceae_bacterium	13	9	0	0	1	0	0	0	1	0	0	0	0	0	0	0	3	0	0	0
Parvimonas	0	0	0	0	0	0	0	0	0	0	0	0	0	0	0	0	0	0	0	0
Ezakiella	5	7	0	0	0	0	0	0	0	0	0	1	0	0	0	0	0	0	0	0
Gardnerella	7	3	12	0	0	0	0	0	0	0	0	0	0	0	0	0	0	0	0	0
Lachnospiraceae_bacterium_feline_oral_taxon_	0	0	0	0	0	0	0	0	0	0	0	0	0	0	0	0	0	0	0	0
